# Selections and Abstracts

**Published:** 1903-03

**Authors:** 


### SELECTIONS AND ABSTRACTS.

---

### SOME OF THE PRINCIPLES WHICH SHOULD GOVERN US IN FRACTURE THERAPY.*

In order that we may accomplish the best results in the management of those complications arising contemporaneously with treatment or succeeding it, it is well, first of all, to direct attention to the prevention of them, to prophylaxis.

And, here, let us first take notice of the unaided powers of Nature in the work of restitution, to note that the tendency of broken bones is to unite very rapidly if left alone. Mr. John Chiene, of Edinburgh, in his address on Surgery, at the meeting of the British Medical Association, at Bournemouth, in 1890, called attention to this and noted as an example costal fracture, which cannot be immobilized and yet unites quicker than any other fracture.

Sampson Gamgee was the first to cast aside the views of pathologists up to his time, on the pathogenesis of fracture, and declared that noncomplicated fracture always united without callus when there was no displacement, and no retentive apparatus was applied that would embarrass the circulation; that bone tissue, as any other, united by primary and secondary union, a fact which has been repeatedly demonstrated in my own experience in hospital and private practice. He called attention to the fact that, in the lower animals, broken bones, without any treatment, quickly repaired, and seldom with deformity.

Lucas Championnière, in his late treatise, has gone so far as to discard all descriptions of mechanical appliances in fracture, except in those cases attended with marked and recurring displacement.

The fear of the law, of the risk entailed by a departure from antiquated methods, professional hostility, rivalry and other reasons often induce practitioners to pause before they take full advantage of the physiological auxiliaries of the economy; furthermore, the

*By Thomas H. Manley, Ph.D., M.D., of New York City, Visiting Surgeon to the Harlem and Metropolitan Hospitals time-out-of-mind custom of splinting every description of fracture has led the laity to suspect that there can be no such thing as fracture unless it is "set" and fixed in mechanical adjustments. But none of those unimportant objections should be permitted to impede progressive science or swerve a conscientious surgeon from his line of duty.

### REST AND MUSCULAR RELAXATION.

The *first step* in the treatment of a fractured limb is to place it in a comfortable position and to guard carefully against inflicting further damage on an already crippled circulation. That attitude of the limb which favors muscular relaxation should be preferred; the flexed position eases the strain on the joints, the extensor muscles and the "false joint" at the seat of fracture. This affords rest of the crushed parts and tends to dispel the shock to the nervous system, with that painful, disquieting apprehension so marked in all severe fractures.

My practice has been so to place broken limbs until muscular spasm has passed off, until the circulation is fully re-established and the acute swelling which accompanies reaction has subsided.

In complicated fractures involving the articulations, those impacted or co-existent with dislocation, the advantages of attitude are not so evident as in those in the shafts involving the articulations, although its adoption is a principle of dominant importance in all.

### THE TIME THESE FRACTURES SHOULD BE PERMANENTLY COAPTATED OR SET.

Circumstances play an important rôle in determining the most appropriate period for the definite adjustment of broken bones, attended with displacement.

It goes without saying that in the exigencies of a military campaign, and under many circumstances in civil life, some kind of immovable adjustment is often imperative immediately after the accident; as, for example, in transporting the injured to their homes or hospitals, or in railway casualties far from hospital stations. Here, however, the adjustment is of a temporary character for short, rapid transport and is permissible, as nothing approaching a precise and exact approximation of the fragments is attempted at the primary dressing. Besides, in very young, restless patients, or in those who reside at so long a distance from a physician that frequent visits are impracticable, there may be justification and necessity for the so-called immediate immobilization.

But, when circumstances admit of it, the immediate, rigid locking-up of every fractured limb in firm splinting is a flagrant violation of one of the most salutary principles of surgery and, without doubt, is responsible for more suffering to the patient, gangrene, delayed or nonunion, stiff joints and wasted limbs than any other factor in fracture-therapy.

A fractured limb is quite invariably, in a limited sense, in a state of suspended animation. Boyer described it as a "torpor of the tissues." The circulation is embarrassed, the normal elements have suffered damage, voluntary control over the muscles is lost, and the limb, for the time, becomes a helpless appendage. In many we will find, immediately after injury, marked, incoercible muscular spasm, this being most notable in those bones moved by or lying among powerful muscles. A good example of this came under my notice recently in a case of patellar fracture, in which a few hours after injury there were nearly five inches of separation of the fragments, while a week later this gap had automatically reduced to less than one inch.

Stimson, in his latest edition on "Fractures," written sixteen years after the appearance of his first superb work, speaks in no ambiguous terms on this phase of fracture treatment, and says: "Generally speaking, the treatment of fracture should begin when the patient is first seen, but by this is meant that every indication should not be at once met by appropriate measures, even the correction of the displacement, the setting of the fracture and the immobilizing of the fragments may have to be left undone or incomplete, because of conflicting and dominating conditions.

"A delay of even several days is usually, in respect of these indications, of small importance; for the preparatory course in the bone and the soft parts goes on notwithstanding it, and, when final adjustment is made, the condition differs but little from that which would have existed had it been made at first. Not every fracture is accompanied with displacement that needs to be corrected, and in those in which displacement exists, in not every one is reduction possible or advisable, and sometimes, when it is possible and advisable, circumstances require that it should be delayed."

Such an admonition from such a source must carry great weight with it, as the author is well known to have had a larger experience in the traumatic surgery of the joints and bones than any other living American surgeon, and has given the profession one of the most complete and valuable works on the subject yet published.

Clearly, Professor Stimson had complicated fractures in mind, or those complicated by internal injuries, although the underlying principle enunciated applies to all.

### ON SPLINTING AND FIXATION OF THE FRAGMENTS.

We are in possession of ample clinical and experimental evidence sufficient to prove that the less compression or tension employed on a limb immediately after injury, the better for aseptic repair and reestablishment of full function.

The mistake is too often made of concentrating one's whole attention on the displacement of the fragments, to the exclusion of those vital and necessary structures, without the full nutrition of which all our efforts are in vain.

Fractures without displacement do not call for immediate splinting, though, as a precaution against displacment later, supports are sometimes, though not always, necessary. Complicated fractures of a multiple character, attended with an extensive shattering of bone and damage to the soft parts, are never firmly splinted without seriously imperiling the integrity of the limb. Many of these do surprisingly well without an adjustment of any kind during the first week or two, if they do at any time. Many times to my amazement at first this has been demonstrated. Among a few of the most notable instances was a man who had been struck by the pole of a heavy brewer's wagon, while the horses were running away. Besides a fracture and dislocation of the clavicle, the humerus was broken twice through the surgical neck and just above the elbow; besides, there were four ribs broken on the same side. For some days the circulation in the arm was so feeble that no attempt was made to apply splinting, besides he was an asthmatic patient who could tolerate no sort of support in fixing the shoulder. For three weeks he lay in a semiprone position, the shattered arm and shoulder lying unencumbered on pillows. However, union here was not only rapid, but apposition was excellent and function was well reestablished.

Not long after, a woman in the sixth month of pregnancy was admitted to my service, who had sustained a terrible smash of the skeleton. She had fallen three stories, on to the stone pavement of a back-yard, in falling striking across a beam for clothes-lines, about six feet from the surface. Besides a double fracture of the right humerus and fracture of the clavicle, all the ribs of the thorax on the right side from the third to the tenth were broken and their ends driven into the lung substance. Emphysema spread widely into the superjacent tissues, and a large escape of blood widely distended the pleural cavity.

The degree of cyanosis following was extreme, so that at no time for three weeks could any bracing apparatus be applied. By the time supports could be borne, primary union of the fragments was evident. Ultimately consolidation with very good form and function followed.

In the summer of 1900 a freight conductor was brought into the hospital, who had fallen and was crushed under the trucks of a freight car. At the time of entrance, besides other injuries, his left arm from the shoulder to the wrist was fractured in four places, one fracture close to the anatomical neck of the humerus, one near, but not involving, the elbow joint, one across the coronoid fossa of the ulna and the neck of the radius, and one just above the pronator quadratus. There was so much shattering of bone that movement communicated to any part of the limb produced sensation similar to so many loose bones in a bag.

Notwithstanding all this comminution of bone and crushing of the soft parts, the main blood trunks escaped, and the nerves, though seriously damaged, showed evidence of vitality. For ten days after admission the arm lay buried under a carbolized dressing on a warmed hair pillow. Enormous distention attended inflammatory reaction, the edema extending down to the tips of the fingers. On the tenth day, as inflammatory symptoms began to subside and it was evident that the limb was saved, it was placed in a loose gypsum dressing. Here, too, union has been rapid and with little deformity.

These cases are among the class which has heretofore been designated "simple fracture," *i. e.*, they were closed. The vitality of a limb, as well as its restoration in action and strength, after being broken depends very largely on the movement of the unimpeded blood current, both the arterial and the venous. In all fractures of the epiphyseal ends or the diaphyses the blood-vessels suffer injuries in varying degrees; and it should never be lost sight of that the very first consideration of the surgeon should be, after his patient is permanently settled in his quarters at home or in a hospital, to examine critically into the state of the circulation and be cautious that his manipulations or the injudicious employment of splinting, of needless force of bandaging, do not augment the damage to the vessels or hamper the movement of the blood current.

The tendency of all fractured bones is towards spontaneous reduction and consolidation, when the degree of dislocation is not very great and there is no impaction of the fragments.

It is, therefore, obvious that a retentive apparatus, at best, only provides a support to steady the fragments during necessary movements of the body. Mechanical fixation, strictly speaking, while being our ideal aim, is a myth and an impossibility by splinting alone. Spiking, wiring and clamping the fragments are expedients rarely employed, except in open fractures. Mr. Arbuthnot Lane, an extensive writer on the subject of "Mechanics in Fracture," says "that the supposition that the surgeon is able to restore the broken bone to its normal form, as it is termed, to set the fracture, by manipulation, etc., and to retain it in position by splints and other means, is, except in a few cases of transverse fracture, quite wrong, as the muscular contraction exerts practically no influence in opposing the restoration of the broken bone to its original form" (*Edinburgh Medical Journal*, September, 1901). Mr. Lane is an ardent advocate of operative exploration and immediate mechanical fixation in all fractures attended with marked displacement, and, while, as is well known, exact approximation of the fragments is not indispensable for the securing of fairly good outlines and full function, we must give due weight to the views of one who has widely opened these parts and inspected them at different stages of the injury, with satisfactory results following.

### IMPORTANCE OF AVOIDING THE EMPLOYMENT OF SUCH MECHANICAL RESTRAINTS AS WILL IMPEDE THE FREE CIRCULATION OF THE BLOOD OR INDUCE UNDUE PRESSURE ON THE NERVE-TRUNKS.

Some years ago the writer made a series of experiments with a view to observing the influence of induced fracture on the movements of the blood current, utilizing the frog for that purpose, as, through the transparent webbing of the feet of this amphibious animal, the elements of the blood, its movements and the investing capillaries show with remarkable clearness. The essay embracing this research was presented at the meeting of the Mississippi Valley Medical Association in October, 1895, and need not be detailed here; suffice it to say that my purpose was to note first the effect produced by fracturing a bone-shaft, in various places, through its diaphysis, near its articulations or into them.

Second, to observe the effects which retentive materials produced when so adjusted as to fix the limb.

In every instance, after induced fracture of any description was produced, the velocity of the blood-stream was slackened in the aterioles, and in whole capillary territories the blood-corpuscles came to a standstill.

When a spiral fracture was produced by a twisting movement of the limb or direct violence into a joint, the circulation everywhere ceased, there was complete stasis in all the vessels, large and small.

This temporary arrest of motion was observed to persist for varying intervals, in fractures attended with shattering and other types, a languid current only beginning after twenty-four hours or later.

In the finer capillaries the vasomotor paralysis often lingered until as late as the third day before blood-corpuscles reentered them. There were, besides, territories which remained entirely devoid of circulation; there had been a free diapedesis, with an entire fading away of the capillary outlines.

My next step was to note the immediate effects of splinting or bandage over the sight of fracture after replacement had been effected. It was impossible to employ any permanent retention apparatus, as it was immediately shaken off. I was, therefore, restricted to observation after immediate coaptation of the fragments. It was curious to note here that sufficient tension of the fine cording used to maintain the splints in position invariably produced a complete arrest of movement in all the vessels of the webbing.

Briefly to summarize, from these experiments it was clearly demonstrable and conclusive that the infliction of a trauma sufficient to disorganize a bone or seriously damage a joint (1) usually impeded temporarily or arrested the free flow of blood through the finer vessels; (2) the tendency was toward early reestablishment in most of the vascular areas, when no pressure adjustment was employed; (3) that many of the finer capillary ramifications may remain permanently obliterated; (4) a compression adjustment or immoderate tension on the injured limb tends to vascular stasis and other ulterior pathological changes in the blood itself and the vascular structures.

Animal experimentation of any description has but a relative application to the human body, more particularly when complex physiological and pathological questions are to be solved. Nevertheless, it has an inductive value not to be underestimated. In this instance the deductions from these experiments were quite in harmony with, and are largely confirmed by, clinical observations. They emphasize the fact that in all fractures, especially the complicated, the coincident participation of the vascular structures with traumatic osseous disorganization is obvious, and impresses the necessity of always giving due regard to this fact in the treatment of broken limbs.

It would seem, however, that in some fractures the damage of the great blood-trunks alone is not sufficient to explain the grave nutritive changes in the limb which may follow, as delayed or non-union or wasting of the limb, the gangrene or mortification sometimes following with or without splinting, because it does not appear, according to the testimony of Stimson, that the ligation of the femoral artery materially interferes with union in a fracture of the lower extremity, and Dr. Merrill Ricketts, of Cincinnati, has recently shown that both the femoral artery and vein may be ligated without the least impairment in function and strength of the limb following. What rôle a lesion of the nerves plays in complicated fractures has not yet been determined, but there are sufficient reasons to infer that a crushed or overstrained nerve-trunk calls for caution not to compromise its vitality by anything which may induce a limitation of the fullest distention of its sheath by extravasated blood or the hyperplasia of acute inflammatory action.

Already, since the advent of modern aggressive surgery, operative intervention has been frequently and successfully invoked in a large number of fracture cases, in which the disability resulting and special symptoms present pointed to callous inclusion, from pressure, or even a complete division of the nerve-trunk.

The nerve-trunk possesses the most extraordinary resisting properties in traumatisms, being the last to perish after a grave injury; in fracture it may be seized and strangled in a callus or between two or more fragments, overstretched or be pierced by a spicula; yet the extraordinary rapidity and completeness of repair. Neural complications here open up a new and highly important field for osteoplastic surgery.—*Gaillard's Medical Journal.*

---

### THE TREATMENT OF THE INSANE IN PRIVATE PRACTICE.*

"Truth will prevail" is a true dictum. What is truth is often the stumbling block in medicine, and the especial vagueness of it all in mental disease a mist that should be dissipated by the sunlight of science and common sense.

We need, it seems to me, more exact observations of insanity as seen clinically as well as of pathologic states, the latter frequently

*Read by F. Savary Pearce, M.D., of Philadelphia, at the meeting of the Medical Society of the State of Pennsylvania at Allentown, September 16 to 18, 1902.

the cause of the alienation, but in the smallest number of cases morbid changes are resultant of a diseased mind, insidious, it is true, but existent surely no matter how difficult to see or test for the neuronic change. All will admit the dominance of mind over body and it must affect function and structure of human cells. We therefore need to emphasize also exacting individualization as to etiology of insanity, to find out what practically caused the imbalance. Was it worry alone or toxins or specific disease; or was the physical state of the body just at such a low (vulnerable) ebb that mind was not sustained in coördination and gave way in the first crash? Psychology must be, in other words, better understood by medical men if insanity as a study is to mount higher in the scale of progress occurring in all lines this dawning twentieth century. And through clinical psychiatry we must then learn to know better the malignity of the mental affection, so to speak, and by close study of a single case we shall once in a while strike the keynote of successful treatment of many; and meanwhile fight on with confident hope for a future for mental disease as with other bodily affections.

We certainly need, it seems to me, better judgment as to cases of insanity for asylum treatment and those to be kept under the domain of private care and management; more exact methods of treating cases of mental disease in private practice. It is axiomatic that asylum treatment is desirable for the majority of cases of mental disease of all sorts and in almost every case of chronic insanity; but there are many instances, especially among "well-to-do" people, that would better be treated at home. The selection of these few chronic cases and the many acute cases for home or out-of-door therapeutics, together with the treatment of them, shall be the object of this contribution to practical psychiatry. Optimism, I must confess, is more the burden of my song as I face along with progress in general in nervous and mental disease.

There is running through almost all cases of insanity, it must be insisted, an individuality more or less masked after all—personal characteristics of body and mind persisting. It is the intuition, one might say, that higher scientific quick deduction which the alienist learns through years of dealing with well and sick minds, that permits him often to give early judgment in a case, for example, as to type of insanity that will ultimately dominate the case, etc. This scientific psychiatrist will, as we advance, more and more quickly and accurately learn which case, too, is to be homicidal or suicidal, the latter of which is one of the important aspects to have knowledge of when the subject of keeping the patient out of asylum comes up for consideration, as it constantly does in every case I have to deal with in hospital, private and consultation work.

The physician's responsibility is great, indeed, in mental disease. His full duty is clear, however: that he should try to keep mentally sick people in normal surroundings. What are the cases that can best be? In the first place, all cases of alienation in which hysteria enters as an element must be carefully selected as among the cases that should be kept out of asylum. For if this substratum is cured the patient does seem to come more quickly to see his mental defect in clearer perspective and will, with the aid of the physician, be able to reason himself out of a mild subacute delusional state which the hysteria has intensely aggravated. So with that more vague hysterical insanity itself—such cases should almost never be placed in asylum among other diseased minds. They can best be treated in private under rest-cure with wise surveillance of an experienced nurse, sympathetically shown by example and instruction, to the overcoming the lack of will that prevails; or even they should be forced to do what is known upon physical examination to be within the compass of their strength—to thus reinforce nerve energy the while you are overfeeding your patient and giving passive exercise by that delightful, energizing adjunct, massage and Swedish movements. In a paper* read before the College of Physicians of Philadelphia last December, these views are gone over in detail and need not be rehearsed here. Suffice it to say that vacillation of symptoms, as in the general diagnosis of hysteria, is important for determination of the coexistence of a hysteria. It being found then in my experience, the riddle is half solved, for the vast majority of such patients recover. Were they placed in asylum the stigma to them would be greatly intensified

* Association of Hysteria with Insanity. Journal of Nervous and Mental Diseases, March, 1902.

and a permanent dementia is very likely to remain. As a *dernier ressort*, however, I would place such a case in asylum where the last effort had been spent, hoping that repugnance to incarceration would arouse a latent consciousness by the mere idea of confinement, vague as that suggestion is to us.

A type of insanity (a delusional depressive type) in my judgment best treated in private or in a hospital for nervous disease are those subacute cases in woman at or near the climacteric who have been overburdened by the cares of rearing a large family of children, who lose interest in household affairs, generally with excitable periods, lasting a few days at a time, in which some member of the family is especially studied in dislike, and he is often threatened openly (these are the cases that usually do not kill anybody). Such imbalance under private care, away from her children, forced feeding, massage, hyoscine in appropriate dosage for the excitable periods, are usually favorable in prognosis and curable, if a good family history obtains, in two to four months. Such an one should surely recover, as in the case of hysterical insanity referred to me in A. B. reported in the paper mentioned. This woman has since then fully recovered after a course of semi-rest treatment, in which *morale*, forced feeding with the stomach tube, and when a faint mental gain had been shown by a partial willingness not to resist us, was added to by indeed restoring her to private (her home) in Delaware, where she made a rapid recovery to the surprise of her home people. We got the good-will of this woman finally, which paved the way to cure.

For want of more scientific classification the hybrid melancholia-hypochondriac cases, the termination of sexual neurasthenia in men, where the idea has become fixed of self-degradation, can frequently be helped better at home by advising and insisting upon social reinstatement through placing them in the good company of interested congenial friends rather than in an asylum. Such a case was seen in Ohio the past summer; the man was advised to be removed from the Massalon Institution to his home; the change was marvelous for the better within a few weeks. This man had been treated as an incurable by his physicians, who were the first to acknowledge the therapeutic advantage of the régime outlined. These cases are not sufficiently studied, we persist. The sight of cases worse than themselves as frequently works to the introspective idea that they are as hopelessly insane as the cases about them, and we do an injustice to mental medicine to permit them to become cases of secondary dementia in an asylum, as well as an injustice to the State. Asylum superintendents are not to blame so much for this state of overcrowding of their hospitals as we in private practice upon whom the burden of blame through commitment must fall.

Hebephrenias, and especially the acute insanities of childhood, generally are also better treated in private or in psycopathic hospitals rather than in asylums. The impressionistic age of youth is much more influenced by other insane persons, and if they are to be at all restored in mind, by all means give a thorough trial to out-of-door treatment. Often the removal of some imaginary foe from a home or too sympathetic a relative, or if not feasible, the restraint in a general hospital, will be sufficient to attain a start toward mental equilibrium, though it may take months to acquire. Persistence, which the busy doctor does not take time to act upon, will very frequently win success. Some of my own graver mistakes in prognosis have been among children.

Mania from overwork, or of a religious nature, can be hopeful of cure by ordinary methods of restraint at home, if the family are first pacified by lending them the confidence of hope, no matter how demonstrative the patient. It is often that the excited relatives are singularly enough the first to urge the physician to commit such a case, they themselves being in mortal terror of a case of insanity. In such instances the physician is on the two horns of a dilemma indeed.

The judicious use of wristlets and anklets to control the patient from harming himself or others, together with full bromidism by mouth, or combined with chloral per rectum, hydrotherapy if temperature exists, will as often surprise the family with the hopefulness of cure of their much-ostracised patient.

### CONCLUSIONS.

For us all the lesson of acute insanities should be modified and retaught, and the earliest possible treatment instituted; to greatly modify all towards the cure of the acutely insane in private, long before there is any occasion to think of asylum treatment, valuable as that is in chronic insanity, and for the homicidal manias and melancholia.

Acute insanities of hybrid types, *i. e.*, those that savor of two or more types, are the most hopeful in prognosis, and are therefore the ones to be kept out of asylums to the last effort. Especially where hysteria enters as an element in the case the patient should be kept out of an asylum.

The physician's duty is to overcome the terror among lay folk for the insane, and to engender that hopefulness of treatment in private as is so with general medical affections.—*Pennsylvania Medical Journal.*

---

WHILE the "Kaiser Wilhelm Der Grosse" was making her way to the port of New York recently, one of the passengers was taken ill with symptoms of appendicitis. An operation was indicated, but as land was so near, it was deemed advisable to wait until the ship arrived. Accordingly, a wireless message was sent while the boat was still fifty miles away, and in response to the call an ambulance was in waiting when the pier was reached. The patient, a son of former Prime Minister Di Rudini, of Italy, was taken to a hospital, operated upon, and is now convalescent.

---

SEVERAL interesting cases of hysteria in animals are related by M. Lepinay, a French veterinary surgeon, quoted in *Philadelphia Medical Journal.* One of them referred to a canary bird which, while singing in its cage, was frightened by a prowling cat. Complete aphonia resulted and lasted for six weeks. The hysterical stigma then disappeared suddenly, just as in the human subject, and the bird resumed its singing.

